# The Book World of Medicine and Science

**Published:** 1906-11-03

**Authors:** 


### THE BOOK WORLD OF MEDICINE AND SCIENCE.

THE RISE AND PROGRESS OF HYDROPATHY IN ENGLAND. By RICHARD METCALFE. (London : Simpkin, Marshall and Co. 1906. Pp. 307. Illustrations 21. Price 3s. 6d.)

THIS book presents us with a fairly good *résumé* of the rise and progress and, we may add, the decline of hydropathy in the British Islands. There are also chapters on the subject relating to our Colonies and to the Continent.

There was a time when hydropathy was extremely fashionable; when numerous diseases of the most opposite natures were supposed to be cured or alleviated by the treatment; when hydropathic establishments sprang up in many directions, and when somewhat extravagant claims were advanced concerning the cures effected therein. Some of these establishments were managed by men of undoubted ability, and others, it is feared, by men of whom so much could not be said. Nowadays the treatment is reserved for those diseases in which time has proved its usefulness.

It is certainly rather curious that the physicians of ancient days should have used water in the treatment of pyrexia, and that modern medical men until lately should have neglected it so much; but we doubt whether Mr. Metcalfe's explanation is the true one. It was not so much because the great baths of Imperial Rome had been abused that the Christians neglected them; but rather because early Christianity revived the idea that disease was of Satanic origin, and had to be otherwise dealt with than by hydropathy and medicine.

The author laments the alteration in many of our hydropathic establishments, which alteration he seems to look on as a sort of degeneration; but medical men, whether owners of hydropathic establishments or not, are after all only human, and it is more than doubtful whether any of these establishments would pay if the hotel and amusement elements were to be expelled from them; and if they were not remunerative to the owners it is too much to expect that they should be conducted at a loss. Nor does there seem to be any good reason why these elements should be incompatible with medical treatment. Besides, we are satisfied that there are still some, perhaps many, "hydros" in which enlightened medical treatment is followed. It is but too true, however, that many medical students have left their schools with only the faintest knowledge of balneology and dietetics; and the practitioner has had to pick up a knowledge of these subjects as he goes on, or possibly never pick it up at all. Too often, also, the practitioner looks upon things connected with hydropathy as savouring of quackery; and even Mr. Metcalfe may admit that there was at one time some little justification for the feeling.

To the younger man, who, in point of date, is far removed from the days when "the water treatment" was on everyone's lips, the perusal of this book—which, by the way, is in great part biographical—ought to prove extremely useful, and it may be recommended to him, especially if he will read it in conjunction with Dr. Huggard's fine work on Climate and Balneology. Mr. Metcalfe interlards his chapters with portraits of the leading hydropathists of a past generation. From a medical point of view we think these would have been as well left out.

In the introduction Mr. Metcalfe says that "we Christians of Europe are a dirty generation in comparison with some of the people of Pagan times," and he might have pointed his moral and adorned his tale by coming considerably farther down than those old Pagan days. He might have contrasted the city of Cordova during the Moorish occupation with the city of the present day. In the former it had 900 public baths. Are there nine in existence now?

ANEURYSM OF THE ABDOMINAL AORTA. By F. P. NUNNELEY, D.M., M.A. (London : Baillière, Tindall, and Cox, 1906. Pp. 121. Price 3s. 6d. net.)

ANEURYSM of the abdominal aorta is a malady of which the medical profession as a whole have but a poor grasp. The pathology of the condition is not well understood and its clinical manifestations are very difficult to appreciate. As a consequence, these aneurysms are often overlooked, and more frequently still they are thought to be present in cases when the symptoms are due to some other cause. Therefore we cannot but welcome a contribution which helps to throw light on the subject. The book is divided into two sections. In the first part, commencing with a short historical review, Dr. Nunneley gives, interspersed with personal observations, a statistical study of abdominal aneurysms. His work is based upon thirty-two cases which have passed through the post-mortem room at St. George's Hospital since the year 1841, and upon other published and available statistics. Part II., which from the clinical standpoint is the more valuable portion, contains details of each of the thirty-two cases collected from the records of St. George's Hospital.

It is interesting to note that the majority of cases—73 out of 110—occurred in patients under forty years of age. As to treatment and prognosis, Dr. Nunneley has nothing cheery to say, for he is of opinion that "aneurysm of the aorta is seldom healed by Nature, and very few cases have been affected by treatment, whether medical or surgical." Unfortunately, the material at his disposal has not enabled the author to express any pronounced views upon the part actually played by syphilis and other reputed factors in the causation of aneurysms of the abdominal aorta. And as we still do not know how to prevent or to cure the malady it must remain one of academic rather than practical interest.

---

SUBMUCOUS EXCISION OF DEVIATIONS AND SPURS OF THE NASAL SEPTUM, with a Report of the First 30 Operations. By ST. CLAIR THOMSON, M.D., F.R.C.P., F.R.C.S. Physician for diseases of the Throat in King's College Hospital; Surgeon for diseases of the Nose, Throat, and Ear in the Seamen's Hospital, Greenwich. (London : Cassell and Co. 8vo. Price 1s.)

IN operative surgery, as in other important matters, a careful attention to a number of small details is essential for success. For this reason alone the author's description of his own mode of performing this delicate and admittedly rather difficult operation cannot fail to be of interest to surgeons practising rhinology, whether or no they desire to follow him exactly in surgical technique. Formerly, the treatment of nasal obstruction due to deviation of the nasal septum, especially when associated with hyperplasia, was anything but satisfactory; and for these cases the operation of submucous resection was received with almost universal approval. The few objections to the operation which have been raised are here discussed and answered by the author, who then proceeds to enumerate its advantages, as well as the indications for performing it, and, moreover, the contra-indications. The notes on operated cases which follow serve incidentally to show the effect which nasal obstruction has upon the health and well-being of an individual and the importance of treating it. Finally, it may be observed that a bibliography, such as that in the last pages of this little book, besides being a recognition by an author of the work of others, invariably adds to the value of his own.

